# Men, masculinity, and engagement with treatment as prevention in KwaZulu-Natal, South Africa

**DOI:** 10.1080/09540121.2016.1178953

**Published:** 2016

**Authors:** Jeremiah Chikovore, Natasha Gillespie, Nuala McGrath, Joanna Orne-Gliemann, Thembelihle Zuma

**Affiliations:** a HIV/AIDS, STIs and TB Programme, Human Sciences Research Council (HSRC), Durban, South Africa; b Human and Social Development, HSRC, Durban, South Africa; c Faculty of Medicine, University of Southampton, Southampton, UK; d Faculty of Social, Human and Mathematical Sciences, University of Southampton, Southampton, UK; e Africa Centre for Population Health, University of KwaZulu Natal, Mtubatuba, South Africa; f Research Department of Infection and Population Health, University College London, London, UK; g INSERM, ISPED, Centre INSERM U1219 - Bordeaux Population Health, Université de Bordeaux, Bordeaux, France

**Keywords:** Masculinity, HIV, treatment as prevention, qualitative, South Africa

## Abstract

Men’s poorer engagement with healthcare generally and HIV care specifically, compared to women, is well-described. Within the HIV public health domain, interest is growing in universal test and treat (UTT) strategies. UTT strategies refer to the expansion of antiretroviral therapy (ART) in order to reduce onward transmission and incidence of HIV in a population, through a “treatment as prevention” (TasP). This paper focuses on how masculinity influences engagement with HIV care in the context of an on-going TasP trial. Data were collected in January–November 2013 using 20 in-depth interviews, 10 of them repeated thrice, and 4 focus group discussions, each repeated four times. Analysis combined inductive and deductive approaches for coding and the review and consolidation of emerging themes. The accounts detailed men’s unwillingness to engage with HIV testing and care, seemingly tied to their pursuit of valued masculinity constructs such as having strength and control, being sexually competent, and earning income. Articulated through fears regarding getting an HIV-positive diagnosis, observations that men preferred traditional medicine and that primary health centres were not welcoming to men, descriptions that men used lay measures to ascertain HIV status, and insinuations by men that they were removed from HIV risk, the indisposition to HIV care contrasted markedly with an apparent readiness to test among women. Gendered tensions thus emerged which were amplified in the context where valued masculinity representations were constantly threatened. Amid the tensions, men struggled with disclosing their HIV status, and used various strategies to avoid or postpone disclosing, or disclose indirectly, while women’s ability to access care readily, use condoms, or communicate about HIV appeared similarly curtailed. UTT and TasP promotion should heed and incorporate into policy and health service delivery models the intrapersonal tensions, and the conflict, and poor and indirect communication at the micro-relational levels of couples and families.

## Introduction

Men’s longer delay in seeking healthcare (Emslie, Ridge, Ziebland, & Hunt, 2006; White & Cash, 2003), higher likelihood of late HIV diagnosis (Corbett et al., 2004; Johnson, 2012), and lower likelihood of remaining in care and higher odds of experiencing deteriorating health after HIV diagnosis (Lessells, Mutevedzi, Cooke, & Newell, 2011; McGrath, Lessells, & Newell, 2015) compared to women are increasingly well-described. The health situation of men has been attributed partly to stigma and fear and, more broadly, the pressures that they face to conform to socially valued representations such as having strength, control, agency, and earning capacity, and being competitive and also capable material providers (Chikovore, Hart, Kumwenda, Chipungu, & Corbett, 2015; Chikovore et al., 2014; Emslie et al., 2006; Gough, 2006; Kerfoot & Whitehead, 1998; Lohan, 2007; Skovdal et al., 2011; Willer, Rogalin, Conlon, & Wojnowicz, 2013). There is, furthermore, growing attention to how structural factors shape the masculinity representations and men’s ability to achieve them. In Africa, rising rates of economic inequality, poverty, and intermittent work, alongside widespread HIV-related psychosocial challenges are recognised as having contributed to shaping men’s behaviour and hence health (Adinkrah, 2012; Barker & Ricardo, 2005; Bhana, de Lange, & Mitchell, 2009; Brown, Sorrell, & Raffaelli, 2005; Chikovore et al., 2015; Dageid, Govender, & Gordon, 2012; Hunter, 2005; Lynch, Brouard, & Visser, 2010; Siu, Seeley, & Wight, 2013).

The HIV public health response is turning towards universal test and treat (UTT) strategies, that is, the expansion of antiretroviral therapy (ART) in order to reduce HIV transmission and incidence in a population, through a “treatment as prevention” (TasP) effect. TasP requires that a high proportion of HIV-positive people is tested, is linked promptly to, and retained in care on effective ART, maintains viral suppression through high adherence, and is regularly monitored (Wilson, 2012). It is important to understand how men’s engagement with healthcare, including HIV care, is influenced within this novel context, given also that gender is held to be created and enacted within micro- and macro-level relational settings (Connell, 2012). Using data from one of five trials currently underway in sub-Saharan Africa to evaluate the field efficacy of UTT, and driven partly by an expectation that UTT and TasP might change perspectives and behaviours of men, this paper examines the influence of masculinity on engagement with HIV care within a UTT and TasP trial context.

## Methods

Data were drawn from a qualitative study that was part of a social science research agenda within the ANRS 12249 TasP trial. The trial was undertaken in Hlabisa, a sub-district in KwaZulu-Natal Province, South Africa, with a population of 228,000, an adult HIV prevalence of 29%, and a network of 17 primary health centres (PHCs) that also offer HIV testing and treatment to eligible people. Implementation of the trial took a phased approach, starting with 4 clusters in March 2012, then adding 6 in January 2013, and another 12 in June 2014. Follow-up in all clusters was planned to run until June 2016 (a fuller description of the trial is provided in Orne-Gliemann, 2016).

Data were collected in the four initial trial clusters using in-depth interviews (IDI) and focus group discussions (FGD). Participants were varied to capture diverse perspectives. Four FGDs were constituted as follows: younger people identified by randomly approaching households; traditional healers (THP) identified through their peers; older people recruited with help from a community caregiver [C-CG]; and a “mixed” group – comprising young and old men and women, and two THPs – recruited with assistance from a community member. Fewer men were recruited because they were either absent or unable to commit to participating. Four repeat sessions were conducted per group, each focusing on a specific topic and lasting 45–120 minutes. Venues for FGDs were school and community halls, a community tuckshop, and a faith-based organisation’s premises. For IDIs, 20 participants (10 men, 10 women; age range 17–64) were recruited based on criteria age ≥16 years and residence in the four clusters. Recruitment entailed directly approaching homesteads, or announcing the study in the waiting area of one of the trial clinics before engaging interested potential participants individually. Household-recruited participants’ HIV status was unknown unless it had, without solicitation, been disclosed during an interview. Clinic-recruited participants’ HIV status was known. Initial interviews were done in January–March 2013 and lasted 30–60 minutes. Ten of the IDI participants, who were varied by distance from health facility, gender, and HIV status, were selected to participate in two consecutive repeat interviews each in July–November 2013. Venues for IDIs were participants’ homes or TasP clinics. An overview of the sample and research process is presented in Table 1, and additional details about the sample are provided in a related paper (Orne-Gliemann, 2016).

A social scientist fluent in the local language facilitated the FGDs and conducted the interviews, and these were recorded, transcribed, translated into English, and then analysed collaboratively by two independent social scientists. Transcripts were entered into Nvivo v10 qualitative data analysis software (QSR, Melbourne, Australia), and coded inductively and deductively. The coding frame was developed initially from three thematic areas of prior interest, namely barriers to HIV testing, acceptability of early ART for treatment and prevention, and partner influence on beliefs and practices. Codes were then refined by being iteratively broken down and reconnected through theoretical coding to form higher-level thematic categories (Morgan, Krueger, & King, 1998; Strauss & Corbin, 1990). Emerging themes were consolidated through re-reading and contextually interpreting data in the transcripts until saturation (Marshall, 1996) was achieved. Issues pertaining to gender relations and masculinity emerged as a central theme, and given prior knowledge that masculinity affects engagement with healthcare, we were then curious to explore further the manifestation of this relationship in the novel context of a UTT and TasP trial. Data pertaining to this theme were pulled out and read further to identify salient sub-themes, which are presented in this paper and illustrated with supporting quotes. The Biomedical Research Ethics Committee of the University of KwaZulu-Natal approved the study (Approval Number BCF104/11) as part of the trial’s social science programme. Informed consent was obtained from each participant, and confidentiality and anonymity were ensured.

## Results

### Men’s unwillingness to engage with HIV care

Accounts both from the men themselves but also from the women detailed how men poorly engaged with healthcare. Men were consistently described as reluctant to test for HIV for fear they might test HIV-positive and “know…when I’ll die or what will kill me” (IDI-R2, Man, 35, unknown HIV status). They were said to rely on “feeling” the body’s health, or scrutinising using the naked eye to try and ascertain their own or their partners’ health status. A 64-year-old man stressed that he did not need to test since he was “healthy”; he, however, would accept home-based testing if healthcare workers discerned symptoms that they deemed warranted testing. Other participants said:

I don’t like testing… There’s no need…I feel healthy …If I test and find that I’m HIV-positive, then what? I become aware of my status and take treatment!…I’d rather not know…(IDI-12, Man, 21, unknown HIV status)

We have sex without a condom because we’re both looking healthy. We say: “See how *fresh* she looks! Just how could I use a condom?” (Man, youth FGD)

Repeated reference was made in the accounts to men’s greater preference of traditional medicine over formal healthcare compared to women. Men seemed to also distance themselves from both HIV and the feminine environment at PHCs when they expressed concern about the domination of women, supposedly also accessing HIV services, at these centres:

At clinic X, there’s a container. Its purpose is known. Usually girls are queueing and it’s difficult for a man to go there as he would find himself asking, “What really am I doing at an HIV clinic?” (Man, youth FGD)

Besides men being said to routinely infer their HIV status from their female partners’ results as they avoided testing (“saying because she’s negative, so am I” [Woman, mixed FGD]), the accounts continued to portray some distancing themselves from HIV. The men argued that even though they had lived precarious lives in big cities, it was prior to the HIV era. They had, they said, since moved away from the cities and, furthermore, adopted what they presumed were safer sexual lifestyles.

These diseases are new…had it been during our days it would have been *bad*, because we were living in big cities.…Life wasn’t that *fast* but it would occasionally happen that you would find someone (a girlfriend)…but now we’re no longer active…(Man, older FGD)

It also emerged that the prospect of blame and rejection meant that men might prefer not to disclose an HIV-positive status before knowing about their partner’s. Related to this, disclosure seemed harder when it was considered after intimacy had already commenced or occurred. A young HIV-negative man said he would not disclose HIV-positive status to a partner because she could spread the news around; rather, he would consider abstaining “until I get someone who I will disclose to before we start a sexual relationship”. Illustrating the rejection faced by partners who are suspected or known to be HIV-positive, a woman recalled her previous encounter with her estranged but since deceased husband thus:

He had lost weight when I met him…I avoided him when he tried talking to me. He remarked that I was looking healthy, that he was thin and dying. His complexion had darkened; his trousers didn’t fit.…I couldn’t stand how he looked. (IDI-R6, Woman, 57, no HIV)

### Gendered tensions around HIV testing

With men being disinclined to test, when women undertook an HIV test and returned with a confirmatory result, men apparently reacted strongly to suggestions to test as a couple or use protection. A 35-year-old woman living with HIV (LWH) described how she bickered with her husband to a point where he discontinued his financial support to their children. Another woman of similar age said she struggled to disclose to her husband, and even though they used condoms, he refused to test until it was too late, and had eventually died. Other participants said:

I asked to go together so he would hear for himself…He said: “every individual knows how they live their life; don’t tell me to go and test because it is you who knows how you live.” (IDI, Woman, 51, LWH, Government patient)

I can openly take treatment but he’ll still declare he’s *negative*. I may even suggest using condoms but he’ll say I should use with other men, and not him. I cannot refuse…because he’ll think that I’m cheating on him. So I’ll continue harming myself against my conscience. (Woman, mixed FGD)

Women said men went as far as accusing them of sourcing the infection, adding to their fears about testing first. They felt they were unfairly blamed as men acquired infection during labour migration as well as through their own behaviour.

P7: Our men would be in Johannesburg for months while we remained at home with children. Sometimes they weren’t even working, but chasing after women. We stayed at home because they fed us; that’s all we cared about. So when he asks what kind of woman I am to have such an illness, I’ll tell him he brought it. (Woman, ∼65, mixed FGD)

P3: He’ll deny bringing it, and demand that you say who infected you instead … Only after a family meeting is called do elders force him to admit …(Woman, ∼65, mixed FGD)

Amid the tensions, women faced difficulty broaching news of an HIV-positive diagnosis, leaving some looking to antenatal care (ANC) for an indirect yet safer channel of disclosure.

One can fear disclosing to her husband, but becomes able to after the ANC visit…It may be the way to disclose…(Woman, older FGD)

Men admitted they were largely responsible for transmitting HIV due, as women had also mentioned, to their sexual behaviour (“I have personally driven a taxi.…It’s true that taxi drivers are cheaters and…they come home and have sex without condoms” [Man, youth FGD]) but also their delay in entering into care. A man lamented the manner in which he had persisted with taking traditional medicine even though he suspected he was HIV-positive. When he was eventually tested following deteriorating health, he still delayed initiating treatment because, by his own admission, he was in denial. Sounding contrite, the man said:

At times I felt like confessing to people that she’s so sick now because of me. When we started our relationship, I could already feel that my health was not good. (IDI-R7, Man, 60, LWH, TasP patient)

It seems that some men might already know their HIV-positive status when women disclose to them. Such men might do one or a combination of behaviours that include “refusing to test”, insisting they are HIV-negative, disclosing indirectly, or trying to avoid conversation about HIV status altogether. A 32-year-old TasP patient narrated how one of her partners initially declared he was HIV-free. When she later disclosed, he feigned disbelief. She had however “seen” ART medication at his house, but he said it was “TB pills”. Her other partner appeared to try to make it clear that he took her disclosure as a form of practical joke; his response was that if she were really on ART, her body would be showing it.

The accounts further revealed that while men’s migration supposedly elevated the chances of acquiring HIV, it also accorded women the much-needed room to test for HIV without their partners’ permission.

…I won’t know what he brings when he returns, but I must still perform my (sexual) duties as a woman. When he’s gone, the fieldworkers visit and ask me to test for HIV, and I won’t need his permission. (Woman, mixed FGD)

Finally, views that men were the ones who infected women, or that they reacted violently when women brought news of HIV infection, were sometimes refuted. A woman LWH described being married to a supportive HIV-negative husband, while another man recounted his personal experience with his partner as follows:

When I was told…I informed my family…and my partner…She went for a test…found out that she was infected.…then told me she was the one who infected me because she had all along known her status…but was scared to tell me. I told her I didn’t mind since I had already found out I was infected. (IDI, Man, 43, LWH, TasP patient)

The couple later had two children before they separated and he entered a new discordant but highly supportive relationship. At the time of interview, he had not directly disclosed to his children, now in their teens, but he felt they knew because he took his medication openly.

## Discussion

Although UTT has the potential to drive accelerated changes within the HIV public health landscape, including positively shifting men’s perceptions and behaviours, this study indicates persisting difficulties among men in accessing and utilising HIV testing, and further illuminates some of the gender-relational dynamics generating or sustaining these difficulties. The accounts displayed remarkable consistency, notwithstanding that the same participants were interviewed in repeat sessions over a period of time. As portrayed in this study, men’s behaviour and perspectives and the wider gender-relational dynamics may be understood in the light of HIV’s well-documented threat to valued masculinity representations such as having strength, sexual competence, independence, and capacity to earn and provide materially (Adinkrah, 2012; Bhana et al., 2009; Chikovore et al., 2015; Connell, 2012; Dageid et al., 2012; Fitzgerald, Collumbien, & Hosegood, 2010; Hunter, 2005; Mfecane, 2012; Roy, 2004; Siu et al., 2013; Skovdal et al., 2011).

In this high HIV prevalence setting where labour-related mobility, which separates couples for lengthy periods while being associated more with reports of risky sexual behaviour (McGrath, Eaton, Newell, & Hosegood, 2015), and poverty and unemployment (Tanser, Bärnighausen, Grapsa, Zaidi, & Newell, 2013) prevail, not only are many men disempowered materially, in addition to being physically incapacitated by illness; they also worry about their ability to oversee and regulate their partners’ sexuality (Chikovore, Lindmark, Nystrom, Mbizvo, & Ahlberg, 2002). According to the masculinity literature, when men feel emasculated, they make an intensified effort to prove their worth (Kimmel, 2004) through actions that may include anger and violence towards women and other men (Bhana et al., 2009; Ratele, 2008). The violence that men direct at women’s initiation of HIV care, or attempts to use protection, thus signals a desire to tighten vigilance over women’s sexuality, in ways similar to that reported for contraceptive use (Chikovore et al., 2002; Moore, Jagwe-Wadda, & Bankole, 2011). One result is that women’s capacity and means to access HIV services or implement other desired health-related behaviours can be curtailed. The woman who confessed to infecting her partner because she had feared disclosing to him illustrates the dilemmas that women experience around being tested first or without their partners’ knowledge (Perez, Zvandaziva, Engelsmann, & Dabis, 2006), and how the failure to communicate facilitates ongoing transmission of HIV.

Instances where dominant portrayals of gender are refuted or circumvented are worth recognising. Although the woman above likely transmitted HIV to her partner – a phenomenon that is also reported elsewhere (De Walque, 2007; Lurie et al., 2003) – it is much more often the case that men are portrayed as the vectors of HIV. Women also described how they accessed HIV care during the times when their partners, who would oppose the seeking of such care, were away migrating for work. Furthermore, it emerged that some HIV-negative men lived with and provided psychosocial support to HIV-infected partners, against views that men react violently when their spouses test HIV-positive. Equally significant and similar to what is described elsewhere in the literature (Fitzgerald et al., 2010) are the difficulties men experience around their own disclosure. The emotionally charged atmosphere among couples, together with family hearings that may be convened in order to assign blame, leaves men wary about testing with their partner as a couple, or openly disclosing should they already know their status. A study elsewhere in rural South Africa reported men avoiding clinic-based HIV couples testing because they saw it as serving to expose them and their sexual behaviour (Tabana et al., 2013).

Our findings draw attention to the micro-relational level where individuals interact; in particular, the tensions and indirect or poor communication within families and among couples, and the actions that individuals openly or discretely take as they pursue their health interests. Literature increasingly highlights the social basis of masculinity, and the heterogeneity of men including their capacity to experience both power and vulnerability at once (Connell, 2008; Dworkin, 2005; Kaufman, 1994). From a wider structural perspective, the late and low marriage rates within the study setting plus the widespread separate couple living (Hosegood, McGrath, & Moultrie, 2009) may be contributing to the poorer intimacy and communication that is observed in this study. Ultimately, the study points to the need for caution to be exercised around treating men as a monolithic and all-powerful category, or interpreting their behaviour without taking into account their own perspectives. Looking at men’s so-called “refusal to test in couple contexts” on the surface may, for instance, conceal the nuances and complexities that underlie this behaviour. As this study indicates, such refusal may in fact be part of the process of disclosing, albeit indirectly.

Men’s absence from clinical trials was also apparent through their low contact rates during this qualitative study. Nevertheless, a sizeable number still participated. Moreover, we employed measures of enhancing rigour that are specific to qualitative research (Lincoln & Guba, 1985; Mays & Pope, 2000), including triangulating data techniques, participants, and analysts (Mays & Pope, 2000), varying participant characteristics (Marshall, 1996), ascertaining congruency within the accounts and between the accounts and literature and theory (Whittemore, Chase, & Mandle, 2001), and holding repeat interviews with the same participants to gauge changes in views over time. We however acknowledge the absence of accounts about men who have sex with men in this study. Their accounts did not emerge within the design that was used, apparently affirming the silence that surrounds this group and its sexuality in these settings (UNAIDS, 2013). However, the group needs to be considered in discussions about the role of masculinity in UTT and TasP.

In conclusion, UTT and TasP promotion should heed and incorporate into policy and health service delivery models the intrapersonal tensions, and more crucially the conflict and poor and indirect communication that occur at the micro-relational levels of couples and families. Testing and re-testing, whether individually or as couples, and disclosure of HIV status need to be promoted and facilitated with sensitivity to men’s (and also women’s) fears and concerns within their relationship, family, and community contexts. Lastly, continuing considerations should be given to how men can be reached in places where they spend time and also feel comfortable.

## Supplementary Material

Appendix

## Figures and Tables

**Table 1:** Description of sample, data collection methods, and issues covered in discussions with participants.

Method	*n* or group (sex: F; M)	Age range	Other characteristics	Serial meetings and number of participants at each meeting	Foci of topic guides for serial meetings
IDI	10 (6; 4)	17–64	5 HIV+; 4 HIV−; 1 HIV status unknown	First (10); second (6); third (5)	*First:* Health problems and care seeking; HIV status awareness; *Second*: Current health status; perceptions and experiences with TasP; use of traditional and other care; stigma and disclosure issues; *Third*: Understanding of TasP; facilitators and barriers to early initiation of ART; current health status and recent care seeking; experiences about TasP: stigma
	10 (4; 6)	20–60	5 HIV+; 2 HIV−; 3 HIV status unknown	First (10); second (4); third (4)	
FGD	Group 1 (7; 2)	∼24–61	All were THP	First (9); second (9); third (8); fourth (7)	*First*: Access to health care in the community; *second*: community and individual experiences and perceptions of UTT; *third*: social support and disclosure; *fourth*: followed a community walk and taking of photos; facilitators and barriers to HIV testing and to treatment initiation and adherence
	Group 2 (10; 1)	∼27–65	Two THP; others had no income source	First (11); second (10); third (9); fourth (9)	
	Group 3 (8; 7)	19–32	2 C-CG; 1 vendor; 1 driver; 1 DW; 10 had no income source+	First (15); second (12); third (11); fourth (7)	
	Group 4 (12; 4)	∼35–70	Seven pensioners; one gardener; one C-CG; seven had no income source	First (16); second (16); third (12); fourth (12)	

Note: HIV+ = HIV-positive; HIV− = HIV-negative; THP = traditional healer; C-CG = community caregiver; DW = domestic worker.
